# Numerous floating oil droplets in the colonoscopy

**DOI:** 10.1093/omcr/omab117

**Published:** 2021-12-11

**Authors:** Yuichi Takano, Atsushi Shirahata, Masatsugu Nagahama

**Affiliations:** 1 Division of Gastroenterology, Department of Internal Medicine, Showa University Fujigaoka Hospital, Yokohama, Kanagawa, Japan; 2 Shirahata Clinic, Yokohama, Kanagawa, Japan

## CASE PRESENTATION

**Figure 1:**
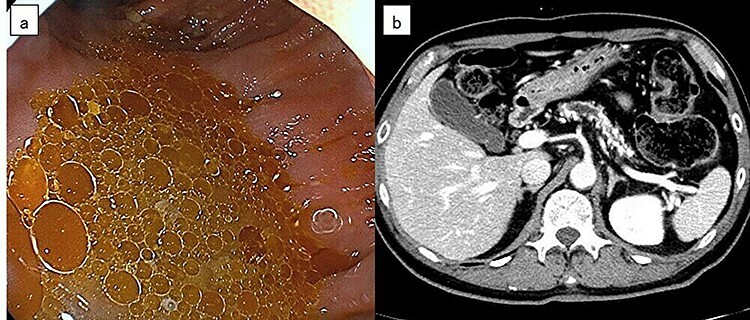
(a) Lower gastrointestinal endoscopy revealed a large amount of oil droplets floating in the large intestine. (b) Abdominal computed tomography showed numerous pancreatic stones in the pancreas and the main pancreatic duct was irregularly dilated. The patient was diagnosed with chronic pancreatitis.

The case was a 59-year-old man who had a 20-year history of heavy alcohol consumption. Watery diarrhea appeared three to five times a day 6 months ago, and a weight loss of 4 kg was observed. Blood tests showed low levels of pancreatic exocrine enzyme; Amy 33 U/L (range: 39–134), Lipase 6 (range; 17–57). A fecal elastase was not measured. Lower gastrointestinal endoscopy was performed. A large amount of oil droplets floating in the large intestine was observed (Fig. 1a).

The findings of colonoscopy were considered to be a fat absorption disorder. Abdominal computed tomography showed numerous pancreatic stones in the pancreas and the main pancreatic duct was irregularly dilated (Fig. 1b). The patient was diagnosed with pancreatic exocrine insufficiency due to chronic pancreatitis.

After starting oral exocrine pancreatic enzyme (pancrelipase granules: 900 mg/day) and a fat-restricted diet (1600 kcal, FAT 40 g/day), chronic diarrhea improved rapidly. Exocrine pancreatic dysfunction should always be in mind as a cause of chronic diarrhea. Oil droplets on lower gastrointestinal endoscopy are a useful diagnostic trigger [1, 2].

## CONFLICT OF INTEREST STATEMENT

The authors have no conflicts of interest to declare.

## FUNDING

None.

## ETHICAL APPROVAL

No ethics committee approval is required for case reports at our institution.

## CONSENT

The patient has given written informed consent for the publication of this manuscript and images.

## GUARANTOR

Yuichi Takano.
